# Fibroelastoma as a Culprit of Syncope

**DOI:** 10.1155/2013/416168

**Published:** 2013-04-15

**Authors:** Giuliano De Portu, L. Connor Nickels, Eike Flach, Latha Ganti Stead

**Affiliations:** Department of Emergency Medicine, University of College of Medicine, 1329 SW 16th Street, P.O. Box 100186, Gainesville, FL 32610-0186, USA

## Abstract

We present a case of a valvular mass diagnosed by emergency department bedside ultrasonography in a young patient with syncope. Bedside ultrasound has become a valuable tool in the evaluation of patients with syncope in the emergency department. This patient was believed to have a fibroelastoma on ultrasound that was confirmed by magnetic resonance and ultimately by postsurgical pathological evaluation. The indications and findings of using ultrasonography as part of the workup of syncope in the emergency department are discussed.

## 1. Introduction

Papillary fibroelastomas (PFE) are the most common tumors of the cardiac valves and the third most common tumors of the heart [1, 2]. Although they are usually not clinically significant and histologically benign, they have been associated with valvular dysfunction, increased risk for embolic events, and even myocardial infarction [1, 3]. We will present the case of a 36-year-old female who suffered a syncopal episode while sitting at her computer. She had no prior episodes of syncope, no systemic signs of illness, and no prior history of intravenous drug abuse. A bedside emergency room ultrasound showed a hyperechoic lesion on the right cusp of the aortic valve concerning for a vegetation, but in this case, it was also concerning for a cardiac tumor.

## 2. Case

**Figure 1:**
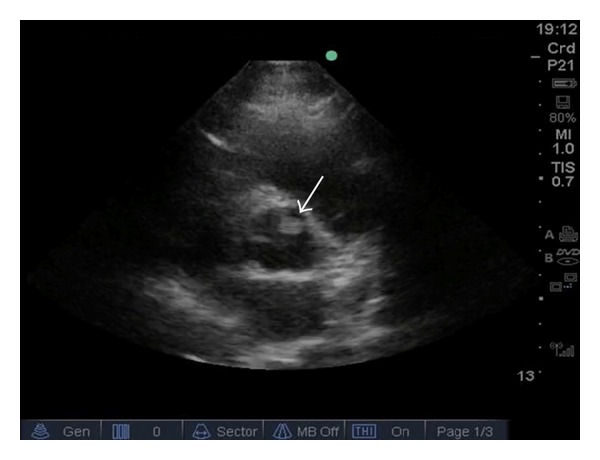
Ultrasound parasternal short axis view showing the aortic valve with the circular well circumscribed hyperechoic area, consistent with a vegetation or valvular mass.

The patient is a 36-year-old female with past medical history of ocular migraines who presented to the emergency department complaining chest pressure and mild shortness of breath. She had a syncopal episode 5 days prior to our hospital visit and was seen and admitted at an outside hospital. Neuroimaging was done as part of her initial syncope workup with negative findings. She signed out against medical advice from the outside hospital, and as she was driving near our facility she developed chest pain. Her family urged her to stop at our emergency department for further evaluation. Patient had never had similar previous chest pain prior to these episodes. On exam, she had intermittent chest pain located mid to left substernal and described as a “constant dull pressure” (up to severity 6/10). She had no worsening or alleviating factors, and her discomfort was nonpleuritic. She had also described dizziness, palpitations, and mild shortness of breath. An electrocardiogram showed sinus rhythm with a rate of 67, and no other abnormalities were noted. Cardiac enzymes were negative. No focal deficits on neurological exam and no cranial nerve deficits were observed. A bedside echocardiogram showed a hyperechoic well-circumscribed lesion on the right cusp of the aortic valve (Figure 1). Differential diagnosis included myxoma, lipoma, vegetation, or thrombus [3].

## 3. Discussion

Syncope is a common emergency room presentation accounting for 3% of total emergency department visits a year [4]. It occurs in the setting of global cerebral hypo-perfusion, and its etiology is due to a variety of reasons that range from the simple to the catastrophic. Typically decreased blood flow to the brain for 6–8 seconds will result in loss of consciousness [4]. * *Costs secondary to admissions for syncope range in the two billion dollars per year [5]. The mission of the emergency medicine physician is to try to identify and “rule out” the life-threatening causes of syncope. The truth is that the emergency department evaluation sometimes will not reveal a clear cause, and many patients will be admitted to the hospital for further workup if they are deemed to be in a high risk group [6, 7]. 

In our case, the patient had never had a syncopal episode and had no other comorbidities. She lived a healthy lifestyle and had no limitations to her daily activities. Her initial workup was negative for any neurological sources that could explain her syncope. On our exam we considered the cardiac causes. Since she had a normal EKG and negative cardiac enzymes we continued our evaluation with the bedside ultrasound exam. “PERC criteria” were used to stratify risk for pulmonary embolism [8]. Patient was low risk for pulmonary embolism and was ruled out by PERC criteria. Blaivas has also shown that unexplained dyspnea could be secondary to a pericardial effusion and can be evaluated and treated in the emergency department [9]. An effusion was not visualized on bedside echocardiography.

The use of bedside ultrasound is useful in identifying other causes of dyspnea and cardiac dysfunction, it has shown to have high sensitivity, and it is fast and non invasive [10]. Emergency physicians use ultrasound with high success during daily evaluations, and it is well established within the American College of Emergency Physician guidelines (ACEP) [10].

**Figure 2:**
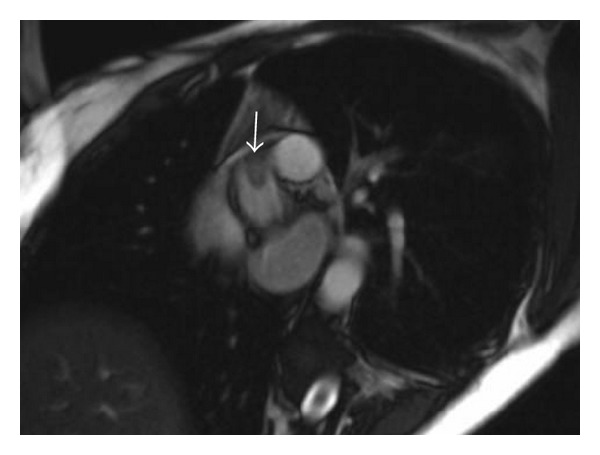
MRI image showing that there is a 1.3 cm pedunculated mass that arises from the right coronary * *cusp of the aortic valve. This mass also demonstrates enhancement on late * *gadolinium-enhanced images and is hyperintense on fat sequences consistent with papillary fibroelastoma.

The patient was admitted to the cardiology service with a consult to cardiothoracic surgery. Further imaging done categorized the lesion as an 1.3 cm aortic valve mass, most consistent with a papillary fibroelastoma arising from the right coronary cusp of the aortic valve (Figure 2). The patient was taken to the operating room, and the surgical team was able to resect the mass completely, sparing the valve, with no other lesions noted and where a transesophageal echocardiogram revealed no residual mass. Patient had also excellent biventricular function, with no aortic stenosis. She recovered well and was discharged with no postsurgical complications. 

## 4. Conclusion

Syncopal workup in the emergency department should include the evaluation of all catastrophic diagnosis. We presented a case of a young female with an aortic mass. Although benign in nature and sometimes only found on autopsy, these are capable of producing syncopal episodes when outflow track is compromised and in some scenarios even neurologic events such as transient ischemic attacks and stroke [11]. It is interesting to note that the majority of patients that present with symptoms are mainly male and with highest prevalence in the 7th to 8th decade of life [12]. The aortic valve is also the most commonly affected [12]. Since the majority of these tumors are located in the left heart, the risk for systemic thromboembolic events is of great concern.

Treatment for symptomatic patients is surgical resection [12]. The tumor is easily removed because of its pedunculated morphology, and as in our patient's case the valve was spared. Recurrence of the mass after surgery has not been reported [13]. Prompt evaluation using noninvasive tools as ultrasound aids in the diagnosis of illness in the emergency department. Emergency physicians are able to make quick decisions about patient care based on those findings. In this case the cause for the syncope was identified, and the surgery team promptly managed the patient appropriately. She is now several months after surgery and has had no other syncopal episodes, and a followup echocardiogram showed no evidence of tumor recurrence.
